# Opium Habit

**Published:** 1879-01

**Authors:** 


### OPIUM HABIT.

Few, except apothecaries, know the extent of the destructive habit of opium eating. Remarkable reports of Asiatic proclivities for the use of this and other poisonous drugs are current in this country, while of the thousands who are wrecked, morally, physically and intellectually by opium, none are generally known, except such as consent to be used as advertisments for nostrum dealers and charlatans.

There seems to be a general impression with the public, which is also indulged by some physicians, that there has been discovered an "antidote," a remedy which can be substituted for opium, used for a short time and then with impunity discontinued. Those who advertise their "opium antidote" are supposed to be in possession of this secret remedy and very extravagant prices are paid for it by the sufferers from the habit.

We took occasion, about a year since, to express our opinion on this subject. We then believed the advertised and dearly sold nostrum to be a humbug—that the cure, when made, is effected by the system of gradual reduction. That it can be done in this way, and in shorter space of time than the nostrum dealers sometimes require, any physician who has tested it will testify. We are induced to take up this subject again in answer to the following note just received:

"——— GEORGIA, Jan. 8, 1879.

DR. J. G. WESTMORELAND:

*My Dear Sir*—Please publish in the next issue of your MEDICAL JOURNAL, the treatment for the opium habit, or give the antidote for the same. Give the full treatment for the cure of those who have been using opium for a number of years.

Yours, ———, M.D."

We do not know how many physicians, like our correspondent, are looking for a substitute by which the opium habit may be cured, but we have reason to believe there are still many who indulge the opinion that some secret drug is being used to satisfy the desire of opium eaters. To such we must express our doubt, at least, of any such discovery having yet been made. It is true that certain other cerebral stimulants, such as alcohol, chloroform, etc., may afford partial relief temporarily, but great suffering and prostration must be the result of sudden and total suspension of the habit, whatever else may be taken.

From analysis of the nostrum "opium antidotes" in use, it seems that strychnine is one of the ingredients. Morphia is of course always found in them, and to the gradual reduction of this, in each successive bottle sent the patient, the cure must be attributed. It is certainly against the pecuniary interest of the nostrum dealer, as well as the feelings of his patient, to reduce the quantity of morphine very rapidly; hence, not very excessive suffering is experienced, but quite a number of bottles, at a high price, are required, and a long while before the cure is effected.

The trouble we find is that our patients often fail to continue honest, fair treatment, when they find in every newspaper certificates and promises of cure by a "painless antidote."

It is not by any means certain that any real benefit is derived from strychnine or any other remedy that has been given with the morphine in order to lessen the necessary quantity. A very ingenious mode of honest cure by gradual reduction has been practiced successfully, as follows: Let a quart solution of morphine in water be made, so that a tablespoonful contains the amount of morphine the patient usually takes at a dose, and let a tablespoonful of the solution be taken at the usual time the habit requires, but after every dose have a tablespoonful of pure water poured into the bottle containing the solution. If the subject is in the habit of taking four grains of morphine twice a day, half an ounce of morphine will make the quart solution of proper strength, and of course less or greater amount may be dissolved, according to the quantity habitually taken. In this way the almost imperceptive reduction effects a cure without excessive suffering.

Another plan is, in addition to moderate reduction of the quantity, to increase the interval between the doses. An hour, or even half an hour's increase of the accustomed interval will very decidedly hasten the cure, but will necessarily give a little more suffering.

Some suffering may be expected by any plan when the cure is effected in a reasonably short time, and the more suffering patients make up their mind to endure, the more speedily they will be free from the habit. Upon faithful observance of the directions all depends, and without unflinching determination on this point the treatment need not be undertaken with the hope of cure.

The cure may be effected in one to three months, according to the quantity taken and the length of time the habit has been indulged.

